# Morphological details contribute to neuronal response variability within the same cell type

**DOI:** 10.3389/fncel.2026.1797436

**Published:** 2026-05-07

**Authors:** Kevin Sandbote, Ihor Arkhypchuk, Jutta Kretzberg

**Affiliations:** 1 Computational Neuroscience, Department of Neuroscience, Faculty VI, Carl von Ossietzky University of Oldenburg, Oldenburg, Germany; 2 Research Center Neurosensory Science, Carl von Ossietzky University of Oldenburg, Oldenburg, Germany

**Keywords:** action potential, compartmental model, Hodgkin-Huxley, invertebrate, ion channels, leech, neuronal excitability, neuronal morphology

## Abstract

Neuronal responses are inherently variable and similar characteristics can arise from multiple combinations of cellular parameters, with electrical diversity and variable branching patterns contributing to degeneracy. The contribution of morphological details, such as the diameter and length of dendritic branches, to response variability and degeneracy in neurons with a given branching pattern remains unclear. We address this question by using a model database approach with spatially extended, conductance-based compartmental models to study the variability of response features, such as resting membrane potential, input resistance, spike count, first spike latency, spike height, and spike width. Using 15 reconstructed morphologies of leech touch cells with fixed branching patterns, we identified thousands of parameter sets that were consistent with the experimentally measured response features in all the tested morphologies. Even when the electrical parameters were kept equal across reconstructed morphologies, variability in response features arose from the morphological details, beyond the well-known dependencies on the total membrane area and input resistance. Varying the spatial distribution of ion channels revealed that spike response features are influenced by the location of spike initiation zones with higher conductance density. Nevertheless, biologically plausible responses can arise from distinct locations of spike initiation zones, or even with a homogeneous distribution of ion channels. Furthermore, comparing the simulated spike responses from two morphological subtypes of leech touch cells revealed that the previously published systematic differences cannot be explained by the morphological differences alone. A larger total conductance of voltage-gated ion channels was required to reproduce the experimental finding of an increased spike count and a larger spike amplitude in the larger morphological subtype. In conclusion, morphological details interact with branching patterns, ion channel distribution and electrical properties, contributing significantly to the variability and degeneracy of neuronal responses.

## 1 Introduction

The brain is often compared to a computer, suggesting that its components exhibit algorithmic functionality and deterministic behavior (Brette, 2022). However, the idea that brains are built of many identical neurons is a fallacy, since cell-to-cell variability in neuronal responses is observed in nervous systems across species, e.g., in hippocampal neurons in rats (Rathour and Narayanan, 2019), the visual system in drosophila (Linneweber et al., 2020), the stomatogastric nervous system of lobsters (Bucher et al., 2005) and the nervous system of leeches (Calabrese et al., 2011; Norris et al., 2011; Roffman et al., 2012; Wenning et al., 2018; Scherer et al., 2022). In addition to this functional response variability, morphological variability in dendritic and axonal structure even within a defined cell type has been reported by many studies (Goodman, 1978; Peng et al., 2021; Moubarak et al., 2022; Meiser et al., 2023). The multiple sources of neuronal variability do not necessarily impair neuronal functionality, because functional network behavior can result from various combinations of neuronal configurations as was shown in experimental and modeling studies (Prinz et al., 2004; Achard and De Schutter, 2006; Alonso and Marder, 2019). The concept of degeneracy, in which distinct combinations of properties lead to similar functional outcomes, has emerged as a key principle exemplifying how biological systems maintain robust performance despite underlying variability (Edelman and Gally, 2001). In the context of neural circuits, degeneracy enables flexible computation and resilience to perturbations (Goaillard and Marder, 2021; Schneider et al., 2023).

Computational modeling has long been a cornerstone of neuroscience research. Modeling approaches, such as the Hodgkin-Huxley formalism (Hodgkin and Huxley, 1952; Almog and Korngreen, 2016), often aimed to identify a single set of “optimal” parameters that reproduced a “typical” experimental observation. While powerful, such models do not always capture the broad range of observed biological variability. Increasingly, studies are adopting a database modeling approach, in which large databases of conductance-based models were systematically examined to demonstrate that diverse combinations of ionic and synaptic properties can generate functionally similar network dynamics and neuronal outputs (Prinz et al., 2003, 2004; Günay et al., 2008; Marder and Taylor, 2011; Jedlicka et al., 2022). These findings led to the understanding of degeneracy and variability, even within cells of the same type. The excitability of an individual neuron is known to depend on its input resistance, which correlates negatively with the total membrane surface area (Kernell, 1966; Kernell and Zwaagstra, 1981) and is also affected by various types of ion channels (Ceballos et al., 2016, 2017). Modern computational studies have focused primarily on variability between different cell types and variability in dendritic branching patterns (Mainen and Sejnowski, 1996; Eyal et al., 2014; Kole and Brette, 2018; Goaillard et al., 2020; Moubarak et al., 2022). However, the role of morphological details beyond cell size and branching patterns in shaping variability in cell-to-cell responses of a specific cell type remains to be explored.

The leech nervous system provides a very accessible platform to investigate this question. It is traditionally considered stereotyped and consists of segmentally repeated ganglia with well-characterized and individually identifiable neurons (Kristan et al., 2005). The mechanosensory touch cell (T cell) encodes light tactile stimulation of the leech skin with its spike count and first spike latency (Pirschel and Kretzberg, 2016). While the same individual T cell responds very precisely to repeated skin stimulation, the responses vary widely between neurons of this cell type (Scherer et al., 2022, 2023). Early studies on leech mechanosensory neurons revealed that T cells exhibit two distinct morphological subtypes, innervating different regions of the skin: T cells with one root process innervating the dorsal area of the skin (1RP) and T cells with two root processes innervating the ventral or lateral area skin (2RP) (Nicholls and Baylor, 1968). A recent study found that these morphological subtypes contribute to the cell-to-cell variability with systematically different response features and speculated that the systematically higher spike counts and larger spike amplitudes observed in 2RP cells are likely caused by a larger area of active membrane compared to 1RP cells (Meiser et al., 2023). This hypothesis cannot be tested experimentally because (to our knowledge) there are no established antibody staining methods for leech neurons capable of marking ion channels or cell structures associated with spike initiation zones (SIZ), as was conducted previously in other species (Meeks and Mennerick, 2007; Guo et al., 2017). Therefore, the distribution of voltage-dependent ion channels across the membrane of T cells can only be inferred indirectly from experimental and modeling studies. Previous studies suggested that T cells have at least two SIZs, one (or more) in their distal processes innervating the skin and one (or more) SIZ proximal to its soma within the ganglion (Burgin and Szczupak, 2003; Kretzberg et al., 2007). Furthermore, a recently published single-compartment model of the T cell was able to reproduce typical response features, but failed to reproduce the experimentally observed first spike latency, suggesting that spikes are not generated in the soma (Meiser et al., 2019).

In this study we use a multi-compartmental modeling approach that incorporates spatial structure and ion channel distribution to analyze their impact on spike generation and to explain systematic differences in response features between the two morphological subtypes. Combining morphological reconstructions, electrophysiological recordings, and conductance-based modeling, we assess how fine-scale morphological differences affect a cell's response to electrical stimulation. Furthermore, our study aims to reveal systematic differences in the response patterns of morphological subtypes of the same cell type. By varying electrical parameters and analyzing responses from a population of reconstructed morphologies, we isolate morphological variability from electrical factors, and clarify its specific contribution to functional variability.

## 2 Results

**Figure 1:**
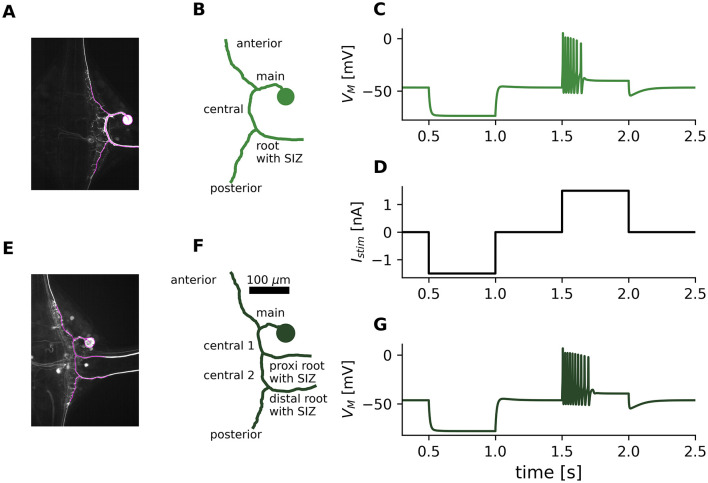
T cell morphologies and simulated responses. **(A)** Image of a 1RP T cell stained with neurobiotin. Magenta lines indicate the part of the morphology that was reconstructed. **(B)** Morphological reconstruction of the leech T cell shown in **(A)**, in which the SIZ was located in the root process in the standard configuration. Diameters of compartments are not drawn to scale here, see reconstruction 5 in Supplementary Figure S1 for a figure version indicating compartment diameters. The main branching pattern consisting of soma, main process, central process, anterior process, posterior process, and one root process, is the same for all 1RP morphologies (Supplementary Figure S1 shows all reconstructed morphologies with their total membrane area and total length). **(C)** Simulated response (obtained with parameter set 2, see Section 4.3) to somatic current injection of this reconstructed T cell morphology. The membrane potential *V*_*M*_ was measured in the soma. **(D)** Stimulus protocol of the current injection *I*_*stim*_ (500 ms long pulses of –1.5 nA and 1.5 nA) into the soma, which was used in experiments and simulations throughout this study. **(E–G)** Cell image, reconstruction and simulated response for a 2RP morphology. See reconstruction 5 in Supplementary Figure S2 for a figure version indicating diameters and the overview of all reconstructed 2RP morphologies. The main branching pattern of all 2RP morpholgies consist of the same processes as the 1RP morphologies, plus an additional proximal root process, which also contains an SIZ in the standard configuration.

Leech T cells usually respond to electrical stimulation of their soma with short bursts of action potentials. However, the number, timing and shape of these action potentials can vary between individual T cells. To investigate the influence of morphological diversity on neuronal response variability, we generated and analyzed a large database of parameter sets that produced biologically plausible responses when applied to the reconstructed morphology of a leech T cell, rather than minimizing specific error values to optimize the compartmental model. We defined a parameter set as *valid* and its model output as *biologically plausible*, if all analyzed response feature values were within the experimentally observed range based on 60 intracellular recordings of T cells taken from (Meiser et al., 2023). Two examples of biologically plausible model responses to the standard electrical stimulation protocol (Figure 1D) are shown in Figures 1C, G. The resting membrane potential and the input resistance were considered as subthreshold response features. The spike features were determined in response to a 500 ms long current injection of +1.5 nA into the soma. These comprised the spike count, the first spike latency after positive current stimulation onset, the spike height, and the repolarization duration, which we defined as the time between the peak of the second spike and the local minimum of the respective afterhyperpolarization (see Section 4.1 for details). All responses were measured in the soma of the cell, in both experimental data and our simulations.

Testing our hypothesis—that morphological details contribute to neuronal response variability within the same cell type—required a multi-step approach. Firstly, we applied a large database of parameter sets to the same reconstructed morphology of a T cell. As quantifying electrical degeneracy would require a comprehensive parameter search within well-defined parameter ranges, which are unknown for the leech T cell, we restricted our analysis to a proof of principle of electrical variability. Using a grid-search on a subset of model parameters, we confirmed that variable electrical properties contributed to response variability and degeneracy consistent with existing literature (Prinz et al., 2004; Achard and De Schutter, 2006; Alonso and Marder, 2019). Secondly, three example parameter sets yielding realistic combinations of response features were applied to a population of 15 reconstructed T cell morphologies that differed only in morphological details. With this approach, we demonstrated the contribution of these morphological details to response feature variability. Since the spatial distribution of ion channels across the membrane of T cells is unknown, we compared different channel distribution configurations in the third step of the analysis to test whether our results depended critically upon the location of the spike initiation zone. Finally, we investigated whether the experimentally observed systematic differences in response features between the two T cell subtypes (1RP and 2RP) could be explained by the morphological differences alone, or whether additional differences in electrical properties were required.

### 2.1 Many parameter sets reproduce T cell responses

Figure 1 introduces our morphological reconstructions. Figures 1A, B show the original microscopic images of two example T cells. The neuron in Figure 1A has one root process (1RP) and the neuron in Figure 1E has two root processes (2RP). To ensure consistent branching patterns and similar total lengths in all reconstructed morphologies of the same subtype (1RP or 2RP), all fine branches were removed and the anterior, posterior and root processes were trimmed (see Section 4.2). This procedure reduced the microscopic image stack to the magenta-highlighted sections in Figures 1A, E, resulting in the reconstructed morphologies displayed in Figures 1B, F. These figures also introduce the labels of the morphological sections that are used throughout this study. Supplementary figures S1, S2 demonstrate that the 15 individual morphologies only differ in the morphological details of section lengths and diameters, resulting in similar ranges of total membrane surface area for the 1RP and 2RP subtypes.

**Figure 2:**
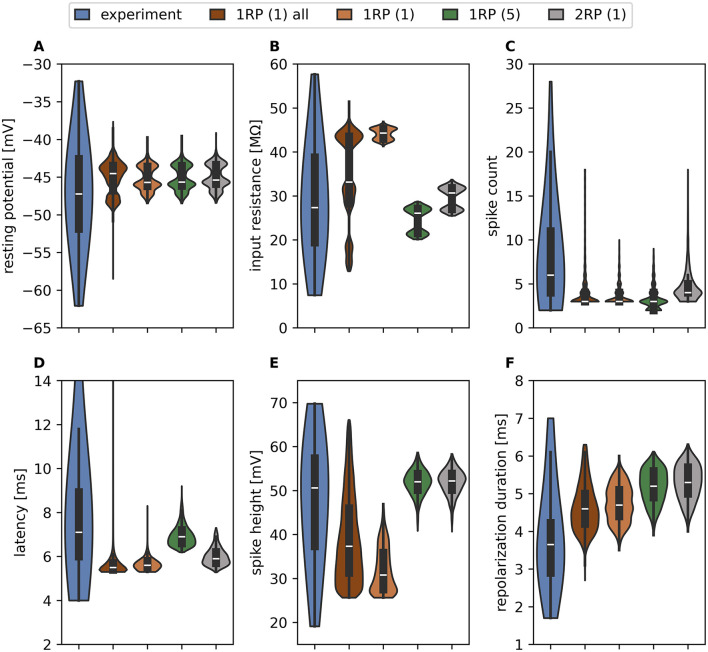
Response variability caused by electrical diversity applied to the same reconstructed morphology. Violin plots and box plots comparing response feature values of experimentally recorded *n* = 60 T cells (blue, data from Meiser et al., 2023) to all valid responses obtained for one example 1RP morphology (*n* = 1,859,170, brown), and responses obtained for the same 1RP morphology with the subset of these parameter sets, that generate valid responses in all morphologies (*n* = 33,958, orange). For comparison, the responses of the reconstructed 1RP morphology (green) and 2RP morphology (gray) from Figure 1 with the same subset of parameter sets are shown. The shown response features are **(A)** resting membrane potential, **(B)** input resistance, **(C)** spike count (in 500 ms), **(D)** first spike latency (4 outliers in experimental data with very long latencies (27.7 ms, 20.3 ms, 16.5 ms, and 15.1 ms) are not shown for improved visibility), **(E)** spike height, **(F)** repolarization duration.

We started the parameter sweep with a single multi-compartment model of a 1RP T cell (morphology 1 in Supplementary Figure S1), placing the SIZ in the single process that innervates the leech's skin. Of the 48,828,125 parameter sets tested for this morphology (all combinations of 11 parameters, each with five values; see Table 1), 1,859,170 yielded valid responses. Comparing the response feature distributions observed in 60 T cell recordings (Figure 2, blue) to those obtained when applying these 1,859,170 parameter sets to the same reconstructed morphology (Figure 2, brown) shows that a large percentage of the experimentally observed range is covered for all six response features. This finding confirms the expectation that electrical degeneracy can account for a large proportion of the experimentally observed response variability. However, the variability in the simulated responses did not cover the entire range observed experimentally for any of the response features. This was expected, given that the electrical parameter space tested was limited to five different values for each conductance and reversal potential parameter and that no ion-channel kinetics were varied. Varying additional electrical parameters could introduce even higher response variability when applied to the same reconstructed morphology.

**Table 1:** Ranges of varied parameters and example parameter sets.

Parameter	Unit	Min	Max	Step	Set 1	Set 2	Set 3
All sections							
*E*_*K*_	mV	–80	–40	10	–50	–60	–50
*E*_*Na*_	mV	20	60	10	50	30	60
*E*_*L*_	mV	–60	0	15	–45	–45	–30
*g*_*L*_	mS/cm^2^	0.02	0.1	0.02	0.1	0.1	0.08
*g*_*K*_*s*__	mS/cm^2^	0.01	0.09	0.02	0.07	0.03	0.07
Passive sections							
*g*_*Na*_	mS/cm^2^	7	11	1	7	8	7
*g*_*K*_*f*__	mS/cm^2^	250	1250	250	750	750	500
*g*_*M*_	mS/cm^2^	1	5	1	1	1	3
Active sections							
*g*_*Na*_(*SIZ*)	mS/cm^2^	140	380	60	140	200	140
*g*_*K*_*f*__(*SIZ*)	mS/cm^2^	600	1140	180	1140	960	960
*g*_*M*_(*SIZ*)	mS/cm^2^	0.1	1.7	0.4	0.5	0.5	0.1

We chose five values for each varied parameter (from Min to Max by Step), resulting in 5^11^ = 48, 828, 125 combinations. The three parameter sets were chosen from the parameter combinations that generated valid responses for all 15 reconstructed morphologies to represent T cells with high (set 1), moderate (set 2), and low (set 3) spike count.

We then applied the valid parameter sets found for one reconstructed morphology to a population of 15 individual reconstructed morphologies (see Supplementary Figures S1, S2). All of these morphologies possess one of the two basic branching patterns shown in Figure 1, RP1 or RP2. Of the 1,859,170 parameter sets, 33,958 (approximately 1.8%) produced valid responses for all reconstructed morphologies. Comparing the brown (results from 1,859,170 parameter sets applied to the example morphology) and orange (results from 33,958 parameter sets applied to the same morphology) response feature distributions in Figure 2 reveals that the range of input resistance was drastically reduced (Figure 2B) and that the range of spike height also decreased considerably (Figure 2E). In contrast, the effects on resting potential (Figure 2A), spike count (Figure 2C), latency (Figure 2D), and repolarization duration (Figure 2F) were primarily observed in the range of outliers.

Applying the 33,958 parameter sets that are valid for the entire population to the two example morphologies shown in Figure 1 illustrates the effects of morphological differences between individual cells. Comparing the response distribution of the first 1RP morphology (orange) with those of the 1RP morphology (green) and the 2RP morphology (gray) in Figure 2 again identifies the input resistance and the spike height as the response features that are most impacted by morphological details (Figures 2B, E). In contrast, the distribution of resting potentials is nearly identical for all three reconstructed morphologies (Figure 2A).

Our results demonstrate that a biologically plausible T cell response can be generated from a multitude of different electrical parameter sets when combined with a single morphological reconstruction. Furthermore, thousands of these parameter sets produced biologically plausible responses for all reconstructed morphologies; the response features nevertheless depend on the specific morphological details. These findings support the idea that T cells exhibit electrical and morphological diversity, both of which contribute to the variability observed in neuronal responses between cells.

### 2.2 Morphological details contribute to response feature variability

**Figure 3:**
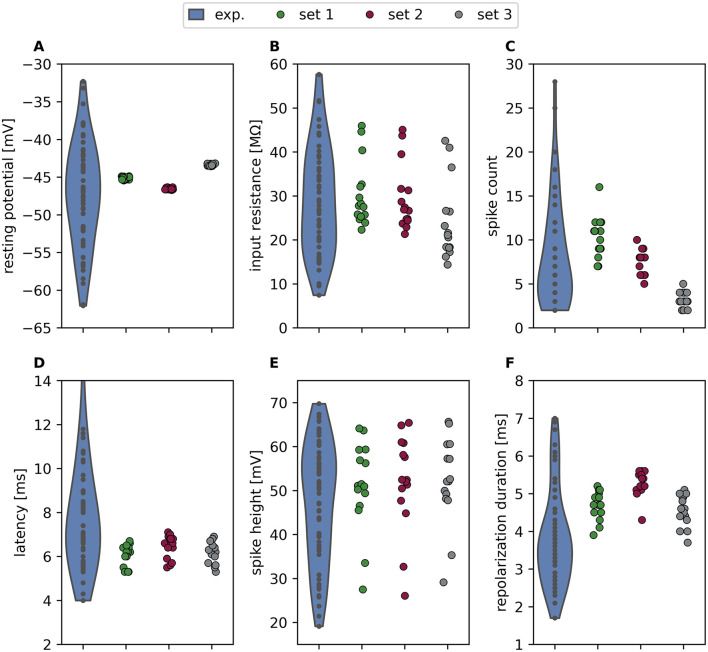
Variability in the responses observed in simulations with the same electrical properties is caused by morphological details. Violin plot of response features of experimentally recorded T cells (*n* = 60, blue, data re-plotted from Figure 2) and strip plots for three example parameter sets (set 1 green, set 2 red and set 3 gray, see Table 1) applied to *n* = 15 different reconstructed morphologies. The range of values shows the variability in response features introduced by morphological details of the reconstructed morphologies. The shown response features (with scaling as in Figure 2) are **(A)** resting membrane potential, **(B)** input resistance, **(C)** spike count (in 500 ms), **(D)** first spike latency, **(E)** spike height, **(F)** repolarization duration.

In the next step of our analysis, we further investigated the response feature variability caused by differences in morphological details. The population of 15 reconstructed morphologies comprised both morphological T cell subtypes, with eight 1RP cells and seven 2RP cells (Supplementary Figures S1, S2). Applying the same parameter set to the entire population enabled us to isolate the impact of morphological details on response variability. We illustrate this approach here using three different parameter sets (see Section 4.3). For example, the model responses to the standard electrical stimulation shown in Figures 1C, G illustrate that the same model parameters (set 2) generate biologically realistic, albeit not identical, responses when applied to two example morphologies. The three parameter sets were selected to produce systematically different spike counts, representing T cells with high (set 1, green), moderate (set 2, red), and low (set 3, gray) activity respectively (Figure 3). To rule out unrealistic combinations of response features (e.g., a very high spike count combined with a very long latency), we compared the experimental data with the model responses using joint feature clouds of all response feature combinations. All of the pairwise combinations of response features obtained by applying the three parameter sets to the 15 reconstructed morphologies fell within the range observed experimentally. This confirms the biological plausibility of the model responses (see Supplementary Figure S3).

As expected from the response feature distributions in Figure 2, morphological details impacted the response features to varying degrees. For the resting membrane potential, the observed range covered only ~1% of the experimentally observed range for each of the three parameter sets. This suggests that cell morphology does not noticeably influence this response feature (Figure 3A). In contrast, the range of input resistance in each of our example sets represented ~50% of the range observed in experiments (Figure 3B). Consistent with the distinct distribution patterns observed for different morphologies in Figure 2B, this result highlights the impact of morphological properties on input resistance. Spike count differed systematically between the three parameter sets and also varied between morphologies within each parameter set (Figure 3C). These findings suggest that electrical differences and morphological variety within a cell population both contribute to the large variability of spike counts observed experimentally. The first spike latencies observed varied only slightly between morphologies and did not differ systematically between the three example parameter sets (Figure 3D). The height of the second spike varied considerably across the reconstructed morphologies, covering ~78% of the observed range in the experimental data, while the influence of the electrical parameters was only marginal (Figure 3E). The repolarization duration was systematically above the median observed in the experiments for all three parameter sets, and differed only slightly between them. Morphological differences had a stronger impact, covering an average of 26% of the range of repolarization durations determined experimentally for each parameter set (Figure 3F).

**Figure 4:**
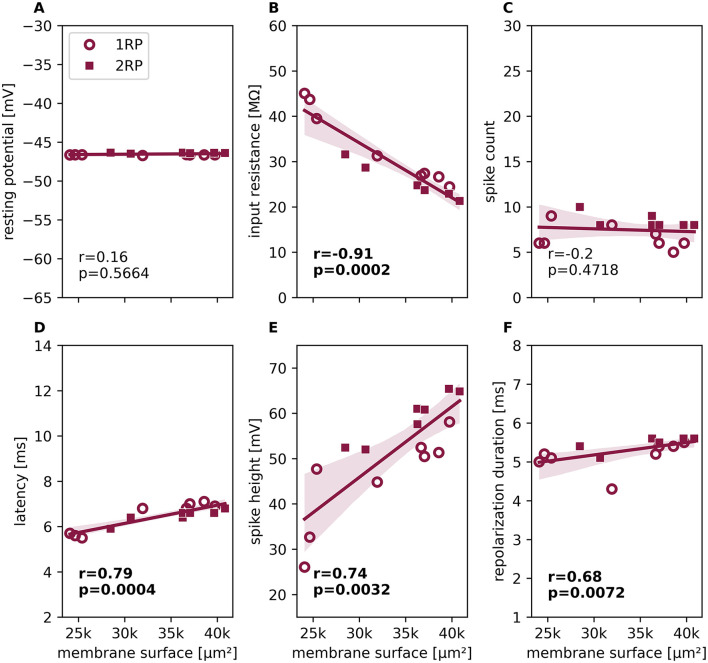
Dependence of response features on total membrane surface area. Response feature values obtained for all *n* = 15 different reconstructed morphologies with parameter set 2, plotted against the total membrane surface area of each reconstructed morphology (see Supplementary Figures S1, S2). Feature values obtained for reconstructed 1RP morphologies are depicted with round symbols, 2RP morphologies with square symbols. Lines show linear regression, shadows indicate the 95% confidence interval. For each response feature, *r* is the spearman correlation coefficient, and the *p* value is given below. Significant correlations are printed in bold. The shown response features (with scaling as in Figure 2) are **(A)** resting membrane potential, **(B)** input resistance, **(C)** spike count (in 500 ms), **(D)** first spike latency, **(E)** spike height, **(F)** repolarization duration.

Next, we investigated whether the variability observed in response features across individual reconstructed morphologies could be explained by the trivial effect of a neuron's total membrane surface area on its input resistance and, consequently, its excitability (Kernell, 1966; Kernell and Zwaagstra, 1981). To control for the effects of cell size, we truncated our reconstructed morphologies to the same distances between the soma and the tips of the anterior and posterior branches, respectively. However, differences in the diameters of individual compartments and segment lengths resulted in a range of total surface areas and lengths for both morphological subtypes (Supplementary Figures S1, S2). Figure 4 illustrates how the response features depend on the total membrane surface area of the 15 reconstructed morphologies for parameter set 2. Remarkably, the total membrane surface areas of the two morphological subtypes (RP1 shown as circles and RP2 as squares in Figure 4) overlapped substantially. Although 2RP morphologies possess an additional root process compared to 1RP morphologies, the total surface area of a 1RP reconstructed morphology with large compartment diameters can exceed that of a 2RP morphology with smaller diameters. While the resting potential was independent of the membrane surface area (Figures 3A, 4A), the input resistance exhibited the expected strong negative linear dependence on the membrane surface area (Figure 4B, *r* = −0.91, *p* = 0.0002). The spike response features latency, spike height and repolarization duration were also significantly correlated with the total membrane surface area (Figures 4D–F). As an additional measure of excitability, we confirmed that rheobase also significantly correlated with the total membrane surface area (Supplementary Figure S4A). However, the most important response feature, spike count, was found to be uncorrelated with the total membrane surface area (Figure 4C, *r* = −0.2, *p* = 0.47). The spike counts obtained for 1RP and for 2RP morphologies appear to scatter randomly around a flat regression line. As all simulations were performed with identical electrical parameters, the considerable deviations of individual data points from the regression line for the spike counts and all other response features must originate from differences in morphological details.

In summary, applying the same parameter set to a population of 15 reconstructed morphologies introduced considerable variability in the responses of T cell models. These differences in response features could not be explained by trivial effects caused by differences in total membrane surface area or systematic differences between the two branching patterns (1RP and 2RP). Overall, these results confirm that morphological details contribute to the observed range of response variability in T cells.

### 2.3 SIZ location influences spike features

As the exact location of spike generation in leech T cells is unknown, we tested whether our results were sensitive to the assumed SIZ location. This was analyzed separately for the two morphological subtypes.

**Figure 5:**
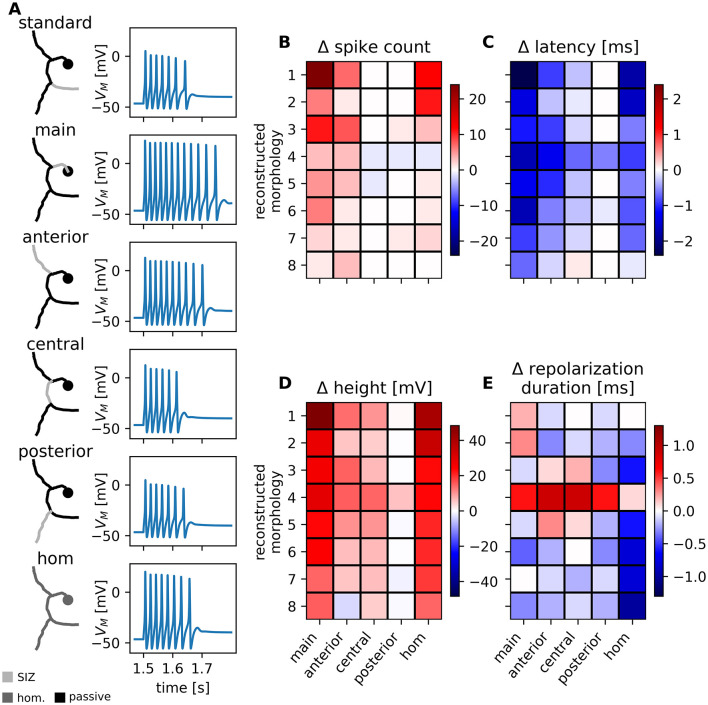
SIZ configuration influences spike response features in all reconstructed 1RP morphologies. Example voltage traces measured in the soma compartment for six different SIZ configurations in the reconstructed 1RP morphology from Figure 1B and heatmaps of spike feature differences depending on the SIZ configuration for 8 reconstructed 1RP morphologies. **(A)** Conductance densities in the membrane of the reconstructed morphology are color coded with light gray indicating higher densities and areas of passive membrane shown darker (see legend). SIZ located in the root process to the skin is the standard 1RP SIZ configuration used in this study. The response features vary when the same total conductance of the SIZ is located in a different cell section (location indicated by the configuration names) or distributed homogeneously over the whole membrane (configuration “hom”). **(B–E)** Heatmaps of spike feature differences depending on the SIZ configuration (x-axes) for 8 reconstructed 1RP morphologies sorted from smallest surface area (1) to largest surface area (8) (y-axes), morphology 5 is the same as shown in **(A)**. Labels of SIZ configurations refer to those in **(A)**. Differences were calculated for each reconstructed morphology by subtracting the response feature value obtained for the standard SIZ configuration from the value obtained for each configuration. The colorbar for each response feature was scaled to the maximum difference calculated among 1RP and 2RP cells. Differences in **(B)** spike counts, **(C)** first spike latency, **(D)** spike height, and **(E)** repolarization duration are color coded with increases shown in red and decreases in blue (see colorbar).

Using the eight reconstructed 1RP morphologies, we tested six SIZ configurations (the SIZ locations are indicated by the bright gray areas in Figure 5A) using example parameter set 2. In the standard configuration, the SIZ with the higher conductance density was located in the root process, while the rest of the membrane had a lower conductance density. The effects of the other SIZ configurations on the spike features are illustrated by the responses of one reconstructed 1RP morphology in Figure 5A. Figures 5B–E quantifies these effects by color-coding the differences between the response feature values of each SIZ configuration (x-axes) and the standard configuration (with the SIZ located in the root process) for each reconstructed 1RP morphology in the population (y-axes correspond to numbers in Supplementary Figure S1, which sorts the reconstructed morphologies by surface area).

Since current stimuli are injected into the soma, where the response features are also recorded, it is logical that the distance between the soma and the SIZ affects the spike response features. When positive current is injected into the soma, the depolarizing effect on the membrane is attenuated with distance. Therefore, it can be expected that the shorter the distance, the more spikes will be generated. The same argument applies to spike amplitude. The shorter the distance a spike travels along the passive membrane, the smaller the attenuation of its amplitude. Since a shorter distance between the soma and the SIZ also causes shorter propagation time, latency decreases. Consequently, the highest increases in spike count and spike height, as well as the greatest decrease in latency, were observed when the SIZ was positioned in the main process, directly adjacent to the soma (configuration “main,” first column in Figures 5B–D). The anterior process is also closer to the soma than the root process, resulting in more, faster and higher spikes for the “anterior” SIZ configuration (second column in Figures 5B–D). The central process is also closer to the soma, leading to the expected decrease in latency and increase in spike amplitude. However, the spike count does not differ from that in the standard configuration for most reconstructed morphologies, since the main process has a larger diameter than the root process (third column in Figures 5B–D). Since the posterior and root processes are at the same distance from the soma and have similar diameters, their spike responses are very similar (fourth column in Figures 5B–D). These results were qualitatively consistent across all eight 1RP morphologies, although the effects differed quantitatively. Morphology 1, which had the smallest surface area, was impacted most strongly by changes in SIZ location. By generating 24 additional spikes with a spike height that increased by 48 mV, it exceeded the range observed experimentally. Unlike the three other spike response features, the repolarization duration did not depend systematically on the distance between the soma and the SIZ. Reconstructed morphology 4, which differs from the others by having a large central process and smaller diameters in the other processes, was the only morphology for which the repolarization duration decreased for all SIZ configurations. The two subthreshold features, membrane potential and input resistance, remained unchanged when SIZ locations were varied (Supplementary Figure S5).

In order to investigate the assumption that T cells possess distinct spike initiation zones with higher conductance density, we calculated the responses of a model in which the total conductance was distributed homogeneously across the entire membrane of the reconstructed morphologies (“hom”). This configuration also produced biologically realistic spike responses. It increased spike amplitude and decreased latency for all morphologies, while the effects on spike count and repolarization period were more variable.

**Figure 6:**
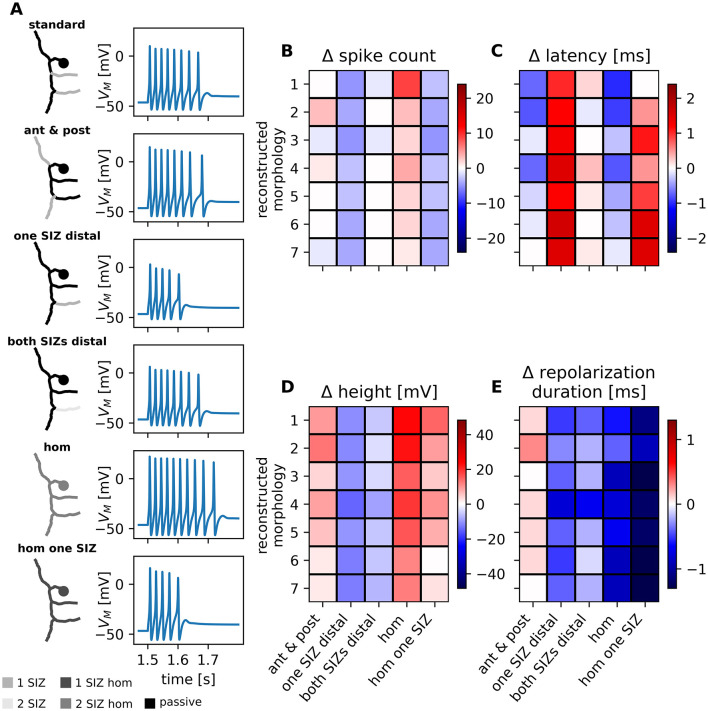
SIZ configuration influences spike response features in all reconstructed 2RP morphologies. Voltage traces resulting from different SIZ configurations in an example reconstructed 2RP morphology, displayed as in Figure 5 and heatmaps of differences in response feature values compared to the 2RP standard configuration. **(A)** Conductance densities in the membrane of the reconstructed morphology are color coded with light gray indicating higher densities and areas of passive membrane shown darker (see legend). Standard 2RP configuration: SIZs located in both processes to the skin. The other configurations are “ant & post”: SIZs with the same total conductance as in the standard configuration are located in the anterior and posterior processes. “one SIZ distal” omits the SIZ in the proximal process to the skin and keeps the SIZ in the other root process as in the standard configuration. “both SIZs distal” combines the conductance of both SIZ in the distal root process. In configuration “hom” the total conductance of both SIZ in the standard configuration is homogeneously distributed across the entire membrane. In configuration “hom one SIZ” the conductance of only one SIZ is homogeneously distributed across the entire membrane. **(B–E)** Heatmaps of differences in response feature values compared to the 2RP standard configuration were calculated accordingly as in Figure 5 for 7 reconstructed morphologies with two root processes and sorted from smallest surface area (1) to largest surface area (7) as in Supplementary Figure S2. The colorbar for each response feature was scaled to the maximum difference calculated among 1RP and 2RP cells. Morphology 5 is the same as shown in **(A)**. Labels of configurations on the y-axis refer to those in **(A)**. Differences in **(B)** spike counts, **(C)** first spike latency, **(D)** spike height, and **(E)** repolarization duration are color coded with increases shown in red and decreases in blue.

For the population of seven 2RP T cells we defined two SIZ, one in each root process, as the standard configuration (Figure 6A, also used in all other figures). This assumption is based on the findings of Meiser et al. (2023) that 2RP cells have systematically higher spike counts and spike heights than 1RP cells. This led them to hypothesize that 2RP cells have two SIZs, whereas 1 RP cells have only one. However, it is generally assumed that most neuronal types have only one SIZ, which is usually located at their axon initial segment (Huang and Rasband, 2018), which is not at a defined location in the complex spatial structure of a leech mechanoreceptor. To test our assumption of two separate SIZs, we tested five additional SIZ configurations for the reconstructed 2RP morphologies and found that these also led to biologically plausible responses (Figure 6).

In one of these configurations (“ant & post”), the two SIZs were placed as far apart as possible in the anterior and posterior processes, without altering the total conductance (Figure 6A). For most morphologies, changing the SIZ location had only a minor impact on the spike count, but resulted in larger spikes, shorter latencies, and longer repolarization periods than in the standard configuration (first column in Figures 6B–E).

With the next two configurations, we omitted the proximal root process. In the “one SIZ distal” configuration (Figure 6A), the reconstructed morphology had approximately half the total conductance of the standard configuration for 2RP morphologies and a similar amount to 1RP morphologies, which, by definition, have only one SIZ. This reduction in total conductance resulted in fewer, much smaller spikes with longer latencies and shorter repolarization periods compared to the standard configuration in all reconstructed 2RP morphologies (second column in Figures 6B–E).

By contrast, when the total conductance of both SIZs was concentrated in a single SIZ in the distal root process (“both SIZs distal” in Figure 6A), the responses resembled those of the standard configuration. The spike count remained consistent across most morphologies, the latency was slightly longer for some morphologies, and the spike height and repolarization period decreased slightly for all morphologies (third column in Figures 6B–E). Consequently, although the “standard” and “both SIZs distal” configurations share the same total conductance, they do not elicit identical spike features. This is probably due to the shorter distances between the soma and the closest SIZ in the “standard” configuration. However, the responses in the “one SIZ distal” configuration differ much more from those in the “standard” configuration, demonstrating the crucial impact of total conductance on spike responses.

To further investigate the impact of total conductance, we considered two configurations in which the total conductance of the SIZs was distributed homogeneously across the membrane of the reconstructed morphologies. Although the “hom”configuration had the same total conductance as the “standard,” “ant & post,” and “both SIZs distal” configurations, it resulted in a higher spike count, larger spike amplitudes, and shorter repolarization periods than any of the configurations previously tested, while the latencies were similar to those of the “ant & post” configuration (fourth column in Figures 6B–E). Therefore, the modeled neurons were more excitable when the voltage-gated channels were distributed across the entire membrane surface than when the same high total amount of conductance was confined to distinct spike initiation zones.

Lastly, in the “hom one SIZ” configuration the total conductance of only one SIZ was distributed homogeneously across the membrane of the reconstructed morphology. This configuration had the same total conductance as the “one SIZ distal” configuration, and approximately half that of all the others. For this homogeneous configuration, we observed as few spikes and similar latencies as we found for the “one SIZ distal” configuration. However, the spikes were much higher and had a much shorter repolarization period than in the “one SIZ distal” and in the standard configuration (last column in (Figures 6–E). Therefore, the responses elicited by the two configurations with the same low total conductance differ more in terms of spike shape than number.

Testing different SIZ configurations for both morphological subtypes revealed that they did not affect the passive response features (input resistance and resting potential, see Supplementary Figure S5). However, the SIZ location and the total conductance influenced all spike features, impacting the individual reconstructed morphologies variably. It should be noted that the parameter sweep was performed based on the assumption of the standard SIZ configurations, combining eight 1RP reconstructed morphologies with one SIZ and seven 2RP reconstructed morphologies with two SIZs. Nevertheless, our results show that biologically plausible responses are not critically dependent on this experimentally untestable assumption and can also be obtained qualitatively with other SIZ configurations. With the exception of the reconstructed morphology with the smallest membrane surface area, the model responses remained within the experimentally observed range for all SIZ configurations and reconstructed morphologies, even when a homogeneous conductance distribution was assumed (Figures 5, 6).

### 2.4 Systematic differences between morphological subtypes can be explained by total conductance of active membrane

The final step of our analysis was to determine whether the higher activity of the 2RP morphological subtype observed in experiments can be explained by morphological differences, or whether a higher total conductance is required.

**Figure 7:**
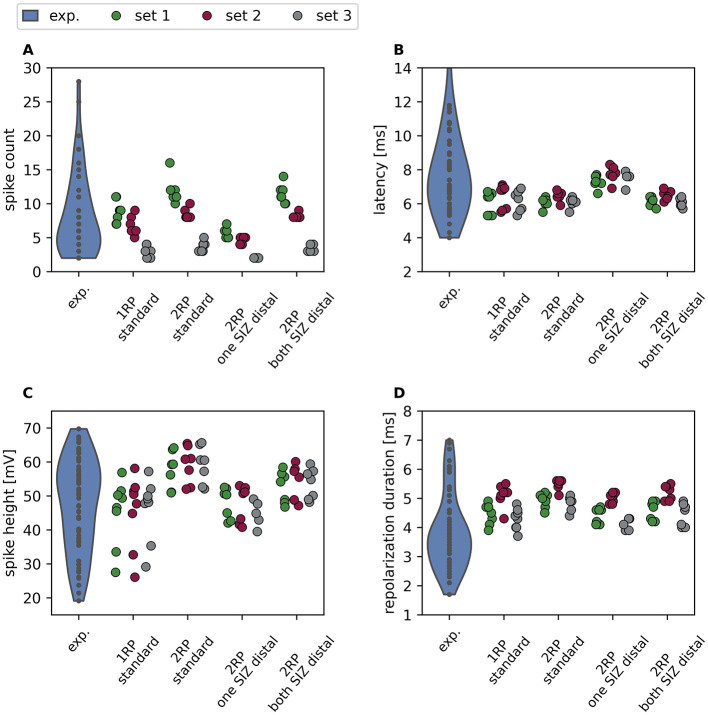
Systematic differences in spike responses between reconstructed 1RP and 2RP morphologies. Violin plot of spike response features of *n* = 60 experimentally recorded neurons (blue, same data as in Figures 2, 3), and strip plots for three example parameter sets (green, red and gray, same parameter sets as in Figure 3) applied to *n* = 8 one root process morphologies in the standard configuration with an SIZ located in their root process (“1RP standard,” see Figure 5A for the standard configuration, and Supplementary Figure S1 for reconstructed morphologies) and *n* = 7 two root process morphologies in the standard configuration with two SIZs in each root process (“2RP standard,” see Figure 6A for the standard configuration, and Supplementary Figure S2 for reconstructed morphologies). Additionally, response features for the 2RP morphologies are shown for the configurations “one SIZ distal” with only one SIZ with approximately half of the total conductance of the standard configuration, and for “both SIZs distal,” in which the full total conductance is located in one SIZ. For each SIZ configuration, the color of the strip plot indicates the applied parameter set. Shown response features are **(A)** spike counts, **(B)** first spike latency, **(C)** spike height and **(D)** repolarization duration.

Figure 7 shows the results from Figure 3 replotted seperately for 1RP and 2RP morphologies with their standard SIZ configuration. All three example parameter sets showed the same trends. In the standard configuration, the 2RP morphologies generated more spikes than the 1RP morphologies when the same parameter set was applied (compare the same colors in Figure 7A). The spikes were also consistently higher in the 2RP morphologies (Figure 7C), while latency (Figure 7B) and repolarization period (Figure 7D) did not differ consistently between 1RP and 2RP morphologies.

To investigate whether the differences in spike count and amplitude are due to the difference between one and two SIZs, we additionally calculated the response features for the reconstructed 2RP morphologies with the SIZ configurations “one SIZ distal” and “both SIZs distal” (Figure 6). When simulating the responses of 2RP morphologies with one SIZ and the same conductance density as the standard 1RP configuration, the spike counts obtained with the “2RP one SIZ distal” configuration were lower than those of the 1RP models with the same parameter set. The spike amplitudes also decreased considerably. However, when the conductance densities of both SIZs were combined in the distal root process (“2RP both SIZ distal” configuration), the 2RP models responded with spike counts similar to those in the standard configuration. The spike heights were intermediate between those of the “2RP standard” and the “2RP one SIZ distal” configurations. These findings suggest that the experimental observation of higher spike counts and amplitudes in the 2RP morphological subtype can be explained by a higher total conductance in 2RP than in 1RP T cell subtypes.

In contrast to the shorter first spike latencies observed experimentally for the 2RP morphological subtype (Meiser et al., 2023), we did not find consistent latency differences between 1RP and 2RP models with standard SIZ configurations in our small sample of reconstructed morphologies (Figure 7B). However, when the SIZ in the proximal root process was omitted in the “2RP one SIZ distal” configuration, the latency increased beyond the values observed for the 1RP models. This would contradict the experimental findings. When the standard total conductance was condensed into one SIZ, we observed latencies similar to those in the standard configuration (Figure 7B), which supports the hypothesis that the 2RP subtype exhibits a higher total conductance.

These results suggest that the previously reported differences in spike features between 1RP and 2RP T cells cannot be explained by morphology alone. These differences include higher spike counts and amplitudes, as well as shorter latencies, in 2RP T cells compared to 1RP T cells. Assuming the same total conductance for both subtypes yields model responses that contradict the experimental findings. Therefore, this modeling study indicates that the 2RP morphological subtype must have a higher total conductance than the 1RP subtype. However, our results do not allow us to draw conclusions about the distribution of ion channels across the membrane surface, as different SIZ configurations lead to responses that are very similar. It is possible that the distribution of ion channels across the neuronal membrane, along with electrical and morphological properties, contribute to the degeneracy of neuronal responses.

## 3 Discussion

Understanding the sources of variability and degeneracy in neuronal responses remains a fundamental challenge in neuroscience (Goaillard et al., 2020). This study demonstrated that, in addition to variability in electrical parameters and branching patterns, variability in morphological details also impacts neuronal response features. Having qualitatively demonstrated the existence of electrical degeneracy for our T cell model by applying a large (though not exhaustive) database of parameter sets to the same reconstructed morphology, we then investigated the impact of morphological details by applying the same electrical parameter set to a population of 15 reconstructed T cell morphologies. Of all the response features, only the resting membrane potential was determined solely by the electrical parameters. In contrast, the input resistance and all spike features were influenced by both electrical parameters and the morphological details of the reconstructed cells. The impact of morphology on the response features was most evident for the spike height and the input resistance: approximately 80% and 50% of the experimentally observed range was covered when identical electrical parameters were applied to only 15 reconstructed morphologies. Response feature variability was partially induced by systematic differences between the two morphological subtypes, alongside variations in total membrane surface area. Nevertheless, even the responses of two morphologies of the same subtype with the same total membrane surface were not identical, demonstrating the impact of the lengths and diameters of individual cellular sections on neuronal responses. Finally, we considered the distribution of conductance densities across the membrane to be a potential additional contributor to neuronal response variability. Although not critical for generating biologically realistic responses, our modeling results show that the location of active membrane affects all spike features. Thereby, ion channel distribution could also contribute to neuronal response variability and degeneracy.

### 3.1 The leech T cell as an example for a cell type with two fixed branching patterns

Leech T cells are a well-studied and experimentally accessible example of neurons that have two morphological subtypes. All T cells are mechanoreceptors that respond to light touch on the skin (Nicholls and Baylor, 1968). Previous experimental and modeling studies have suggested that all T cells require at least two SIZs to reliably transmit signals from the skin to the central part of the ganglion: one peripheral SIZ is located in the skin, and the other SIZ is located close to the ganglion (Burgin and Szczupak, 2003; Kretzberg et al., 2007). Here, we simulated a T cell in an isolated ganglion, disregarding the distal processes that innervate the skin with the peripheral SIZ. Unfortunately, due to experimental limitations, we were unable to measure the distribution of ion channels on the membrane of T cells, which prevented us from determining the exact SIZ location. Electrophysiological recordings only allow indirect reasoning: Since the first spike in response to current injection into the soma occurs at a median latency of 7 ms, the SIZ is unlikely to be close to the soma. This argument is supported by a previous modeling study, in which a one-compartment model failed to reproduce the T cell response latency (Meiser et al., 2019). Our simulation results for the 1RP morphological subtype yielded consistent results. When the SIZ was located directly next to the soma in the main process, the simulated latencies were shorter than those observed in experiments (Figure 6C). However, as several different configurations of channel distributions, including SIZ locations in different segments, as well as homogeneously distributed ion channels, reproduce biologically plausible responses, our approach was unable to determine the location of the SIZ in biological T cells. Our results suggest that the location of the central SIZ is important for spike timing and other spike response features, although this may vary from cell to cell. It is also possible that the location of the SIZ in T cells changes dynamically, thereby contributing to homeostatic plasticity. This mechanism was proposed by prior studies to regulate the excitability of cells in response to sensory or synaptic input (Grubb et al., 2011; Yamada and Kuba, 2016; Goldstein et al., 2019).

Understanding how morphological properties influence neuronal responses is a crucial task in computational neuroscience, especially for classifying and understanding the function of subtypes of neuronal cell types (Chand et al., 2015; Kole and Brette, 2018; Peng et al., 2021; Scala et al., 2021; Moubarak et al., 2022; Mao and Staiger, 2024). In this study, we compared two morphological subtypes that are also known to differ in function. In each ganglion, two T cells of the 1RP subtype innervate the dorsal region of the leech skin, while the remaining four T cells have a 2RP morphology and respond to touch in the ventral and lateral skin regions (Nicholls and Baylor, 1968). The distribution of ion channels across the membrane is even less clear for the 2RP subtype than for the 1RP subtype. Since the root processes of both subtypes reach the skin at different positions, it is reasonable to assume that they both possess a peripheral SIZ that sends action potentials toward the ganglion when the respective skin area is stimulated. It is unclear whether this morphological subtype possesses two central SIZs. However, our simulations show that the experimental observation that T cells with two root processes exhibited higher spike counts and greater spike amplitudes than their single-root counterparts (Meiser et al., 2023) can only be explained by a higher total conductance in the 2RP morphological subtype. Assuming the same total conductance in both subtypes, the larger membrane area of the additional root process in 2RP models reduces their excitability, resulting in fewer spikes being produced than in 1RP models. Therefore, the 2RP morphological subtype must possess a greater number of ion channels. However, our modeling approach cannot determine whether these channels are located in one densely populated SIZ, split between two SIZs, or distributed across a larger membrane area. Due to the lack of specific ion channel markers for leech neurons, the hypotheses derived from our model simulations cannot be tested experimentally in this system. We therefore hope that our study will inspire scientists working with standard model organisms to experimentally measure the variability in ion channel distribution for a cell type with well-defined branching structure and to determine its impact on the variability and degeneracy of neuronal responses.

### 3.2 The interplay of electrical diversity, branching patterns, morphological details and channel distribution as sources of variability and degeneracy

In our modeling study we used leech mechanoreceptors as an example. However, our findings are relevant beyond this specific system because the question of how electrical and morphological diversity lead to variability and redundancy in neuronal responses is fundamental to any nervous system. This question can be demonstrated even with a simple one-compartment integrate-and-fire model that reacts to current injection with spikes when the passive response crosses the spike threshold. A population of one-compartment models with variable leak parameters would generate variable numbers of spikes. The same response variability could be observed in a population of one-compartment models with variable membrane surface area. Combining these two sources of response variability would produce the same number of spikes for multiple combinations of leak and surface area parameters, resulting in degeneracy caused by the interaction of electrical and morphological diversity. In comparison to this simple system, our multi-compartment models of leech T cells have many more degrees of freedom for introducing electrical and morphological variability. For our aim to obtain meaningful results about the impact of morphological details on neuronal response variability, we had to reduce the variability in electrical parameters, channel kinetics, morphological features and spatial distribution of ion channels.

To focus on morphological variability we attempted to minimize electrical variability as much as possible. Therefore, we conducted the majority of the study by applying a single set of electrical parameters to all reconstructed morphologies. To ensure that our results were not limited to this specific choice of electrical parameters, we confirmed them using two additional parameter sets. This minimalist approach allowed us to analyze the interaction between electrical and morphological diversity. We applied a simple grid search to identify example parameter sets that robustly fulfilled our validity criteria across the population of reconstructed morphologies, thereby ensuring that all six response features were within the experimentally observed range. To validate this, we confirmed that the model responses produced by the example parameter sets matched the experimentally observed pairwise combinations of response features (see Supplementary Figure S3), even though the parameter search considered the features independently. This approach was not intended to quantify electrical degeneracy, as this would require a more complete understanding of biologically realistic parameter ranges than is currently available for leech T cells, as well as more sophisticated parameter search strategies, such as the Markov chain Monte Carlo method (Arnaudon et al., 2023), evolutionary algorithms (Reva et al., 2023) or simulation based interference (Tolley et al., 2024). Nevertheless, even this grid search yielded thousands of valid parameter sets, confirming the findings of prior studies on degeneracy in neural systems (Prinz et al., 2004; Achard and De Schutter, 2006; Alonso and Marder, 2019).

Our decision to exclude channel diversity from the scope of the study was the most restrictive aspect of minimizing electrical variability, as this factor is known to play a significant role in neuronal variability and degeneracy (Kole et al., 2007; Berger and Crook, 2015; Goaillard and Marder, 2021; Schneider et al., 2023). While the leech genome contains four Na^+^- and 40 K^+^-channel contigs (Northcutt et al., 2018), the only study of leech neuron transcriptional profiles (Heath-Heckman et al., 2021) did not focus specifically on these channels. Consequently, the typical set of expressed channels and their variability remain unknown for leech T cells. Therefore, rather than modeling specific ion channels, we combined their effects into one fast transient Na^+^-channel and three K^+^-channels (one fast, one slow and one M-type). These were fitted prior to the parameter sweep for one morphology based on our patch clamp recordings (Supplementary Figure S7) and publications on leech neuron conductances (Valkanov and Boev, 1988; Johansen, 1991; Meiser et al., 2019, 2023). When performing the parameter sweep for all morphologies with constant channel kinetics parameters, we found that the repolarization duration was above the median for most of the valid parameter sets and reconstructed morphologies. This suggests that the ion-channel kinetics should be tuned toward a slightly narrower spike shape in future modeling studies of leech T cells.

Since the focus of our study was the variability introduced by morphological details, and since morphological properties such as soma size and location, dendritic arborisation, and axonal properties are known to shape neural response features (Mainen and Sejnowski, 1996; Brette, 2013; Hesse and Schreiber, 2015; Fékété et al., 2021), we took a conservative approach that underestimates the effects of morphology rather than overestimating them. Rather than generating an ensemble of morphologies (Arnaudon et al., 2023; Reva et al., 2023), we strictly relied on the original reconstructed morphologies sampled from microscopy image stacks. The leech T cells enabled us to compare two distinct branching patterns, differing by only one additional root process in the RP2 subtype compared to the RP1 subtype. This clear distinction is advantageous compared to cortical neurons, for which substantial variation in branching patterns was found within the same type (Markram et al., 2015; Arnaudon et al., 2023). Each of the 15 reconstructed morphologies (Supplementary Figures S1, S2) comprised over a hundred interconnected compartments of various diameters and lengths. To focus on the variability introduced by these details, the morphologies were made as comparable as possible in two steps. First, the morphologies were reduced to their main branching pattern, consisting only of the processes that are labeled in Figure 1. All fine branches were removed to prevent variability from additional branching points. In this way, our approach likely underestimated the impact of morphology, since we used simplified reconstructions of morphologies and therefore did not take the fine arborization into consideration, which is also a known source of variability (Cuntz et al., 2010; Peng et al., 2021). Secondly, the reconstructed morphologies were shortened to the same length from the soma to the tip of the process. This compensated for the non-biological variability introduced by our method of dissecting the ganglia in an unstandardized, visually guided way. However, it also neglected differences in the total size of the biological neurons. Despite this rigorous method of standardizing the population of reconstructed morphologies, all response features except the resting membrane potential differed considerably between them. Although total surface areas varied by almost a factor of two due to variable compartment diameters, with substantial overlap between morphological subtypes, response variability could not be explained by total surface area alone. Most response features only exhibited a weak correlation with total membrane surface area, and spike count was uncorrelated. Therefore, it was minor morphological differences, such as the diameter and length of a dendritic branch, that caused variability in the model responses. These complex effects of morphological details likely impact neuronal response variability in general, potentially contributing to variability at the network level (Gowers and Schreiber, 2024).

The last factor in our analysis was the distribution of conductance densities across the membrane. Since ion channel distributions cannot be determined experimentally in leech neurons, we limited the variability by defining a standard configuration which we varied systematically. In the standard configuration, we distinguished between “active sections” in the root processes and “passive sections” everywhere else. The “active section” functioned as an SIZ due to much higher conductance values for Na^+^, slightly higher conductance of the fast K^+^-channel and lower conductance for the M-type fast K^+^-channel, while all other electrical parameters were universal to all compartments (Table 1). First, we shifted the SIZ to different sections while keeping the total conductance constant. This showed that SIZ proximity to the soma can enhance excitability and strongly influenced spike latency. This is in line with findings of a previous modeling study, reporting that spike latency of vertebrate neurons is mostly influenced by cell size and SIZ location (Gulledge and Bravo, 2016). Since spike timing encodes significant information in mechanosensation (Johansson and Birznieks, 2004) our results underline the crucial role of SIZ location in mechanoreceptors. When we distributed the total conductance homogeneously over the entire membrane, we found that distinct SIZ are not strictly needed for the generation of biologically realistic spikes, in agreement with previous modeling studies (Kretzberg et al., 2007; Meiser et al., 2023). For almost all reconstructed morphologies the repolarization period was closer to the median value of experimentally observed responses when a homogeneous channel distribution was assumed. This could suggest that we underestimated the ion-channel conductances in the passive compartments. In the final step of our analysis, we confirmed the hypothesis that the 2RP subtype has a larger total conductance. Our finding suggests an interplay between morphology and electrical properties that explains subtype-specific functional differences but we could not resolve the question whether the additional channels are located in the additional root process, for which no experimental validation is available.

### 3.3 Conclusions

In this study, we focused on the contribution of morphological details to the variability of neuronal response features. This addressed a knowledge gap in the growing field of neuronal response variability and degeneracy, since most studies at the single-neuron level focus on the diversity of channel kinetics (Goaillard and Marder, 2021), electrical properties (Schneider et al., 2023), morphological branching patterns (Mainen and Sejnowski, 1996), or the interaction of these factors (Arnaudon et al., 2023; Reva et al., 2023). Many studies extend beyond the single cell level and study degeneracy in networks of connected neurons (Prinz et al., 2004; Mainen and Sejnowski, 1996), where chemical synaptic (Roffman et al., 2012) and electrical coupling between cells (Crodelle et al., 2019) are also recognized as contributing factors to neuronal variability. Our approach was to minimize all these important factors to focus on the often neglected contribution of diameters and lengths of cellular sections on variability. We found that these morphological details significantly contribute to the variability of the observed response features. As this additional source of diversity interacts with electrical parameters, branching patterns, and channel distribution across the membrane, morphological details may be a relevant yet underestimated factor in experimentally observed response variability and degeneracy.

## 4 Materials and methods

We wrote custom software in Python 3.9.13 for data analysis and simulations. T cell models were implemented using Brian 2, version 2.5.4 (Stimberg et al., 2019).

### 4.1 Data analysis of electrophysiological data

Data from *N* = 60 cells of the previously published T cell dataset from Meiser et al. (2023) were reanalyzed. The test protocol was 11 s long and consisted of a –1.5 nA pulse for 500 ms starting at 4 s and a 1.5 nA pulse for 500 ms starting at 8.5 s (Figure 1C). We only considered the second spike of the response for spike shape features, because the shape of the first spike differs systematically from subsequent spikes. Therefore, we excluded all cell responses with a spike count of 1. We analyzed the following response features:

- Resting membrane potential [mV] was determined as the median membrane potential over 500 ms before the first stimulation.
- Input resistance *R*_*I*_ [MΩ] was calculated as follows:

$$\begin{array}{c} R_{I}=\frac{V_{hyp}-V_{rest}}{I_{stim}} \end{array}$$ (1)

In Equation 1, *V*_*hyp*_ is the median membrane potential during a 500 ms hyperpolarizing current pulse of *I*_*stim*_ = −1.5 nA and *V*_*rest*_ is the resting membrane potential.

- Spike count was defined as the number of action potentials in response to a 500 ms depolarizing current injection of +1.5 nA.
- Latency was determined as the time difference between the stimulus onset of the +1.5 nA current injection and the peak of the first spike.
- Spike height was measured as the absolute voltage difference between the peak of the second spike in response to current injection of +1.5 nA and the minimum of the respective afterhyperpolarization within a time window of 10 ms.
- Repolarization duration was defined as the time difference between the peak of the second spike in response to +1.5 nA current injection and the minimum of the respective after hyperpolarization.

The rheobase was chosen as a measurement of excitability (Rotterman et al., 2021). We applied incrementally increasing current pulse amplitudes from 0.1 nA and 1 nA in steps of 0.1 nA for 500 ms, and determined the rheobase as the lowest amplitude that elicited a spike. Since our experimental stimulus protocol was not designed for a rheobase measurement, we only determined the rheobase for simulated responses (Supplementary Figure S4).

### 4.2 Morphological reconstruction

We used 15 of the anatomical reconstructions previously published by Meiser et al. (2023). Eight were reconstructions of T cells with one root process (1RP), and seven were T cells with two root processes (2RP). The comparison between the microscopy images and the corresponding reconstructed morphologies is shown for two examples in Figure 1. Supplementary Figures S1, S2 display all 15 reconstructed morphologies, and indicate the diameters of their compartments. We applied a Ramer-Douglas-Peucker algorithm to all reconstructions to simplify structures equally and reduce variability in the number of compartments between cells (Hirschmann, 2014). In the algorithm, we set the distance tolerance ϵ to 1 μm for all morphologies, resulting in a median of 120 compartments for 1RP reconstructions and a median of 162 compartments for 2RP models. To reduce methodological variability, the minimum distance from the tip of the anterior process to the soma were determined, and the anterior process of all morphologies was truncated to this length (see magenta lines in Figures 1A, E). The same procedure was applied to the posterior process. Root processes were also cut to the shortest length found in the population to ensure same length across all morphologies.

### 4.3 Multi-compartment model

We reproduced electrical T-cell responses with a conductance-based multi-compartmental model by applying the parameter sets listed in Table 1 to the general Equation 2 and its specifications in Equations 3–10. The dynamics of the membrane voltage in each compartment was calculated as follows:


$$\begin{array}{c} C_{m}\frac{dV}{dt}=I_{K_{f}}+I_{K_{s}}+I_{Na}+I_{M}+I_{L} \end{array}$$ (2)

where *C*_*m*_ = 1 μF/cm^2^ is the standard membrane capacitance, *V* is the membrane voltage at time point *t* and *I*_*x*_ describes the transmembrane current for each current type (see Equations 3–7). Compartments were connected by an intracellular resistance of *R*_*i*_ = 100 Ω cm, as in previous studies (Goethals and Brette, 2020). Voltage clamp experiments in our lab revealed fast and slow components in the K^+^ current (Supplementary Figure S6), so we added corresponding ion-channels in our model. Due to the large size of leech cells, only rough estimates of the ion channel kinetics were possible. These estimates served as a starting point for manual tuning of channel kinetics. For the fast K^+^ current, we implemented a Shaw-like Kv3.1 channel which is often expressed in cells that generate rapid trains of action potentials (Kanemasa et al., 1995). Channels of this type were also identified in the transcriptome of *H. verbana* (Northcutt et al., 2018). We formalized the current of the fast potassium channel as follows:


$$\begin{array}{c} I_{K_{f}}=g_{K_{f}}\cdot n_{f}^{4}\cdot p\cdot \left(E_{K}-V\right) \end{array}$$ (3)

where *g*_*K*_*f*__ is the conductance density of the fast potassium channel, *n*_*f*_ and *p* are the gating variables (see Equations 8, 9), *E*_*K*_ is the reversal potential of K^+^ and *V* is the membrane potential. High conductance densities (Table 1) were necessary to recreate the typically steep repolarization phase of T-cell action potentials. For the slow potassium current we defined:


$$\begin{array}{c} I_{K_{s}}=g_{K_{s}}\cdot n_{s}\cdot \left(E_{K}-V\right) \end{array}$$ (4)

where *g*_*K*_*s*__ is the conductance density for the slow potassium channel and *n*_*s*_ is the gating variable (see Equations 8, 9). This current was introduced to represent the slow component of the K^+^ current, shown in Supplementary Figure S6. This current impacts the afterhyperpolarization and the spike count already with small conductance densities (Table 1). The fast transient sodium channel (Johansen, 1991) was formulated as:


$$\begin{array}{c} I_{Na}=g_{Na}\cdot m^{4}\cdot h\cdot \left(E_{Na}-V\right) \end{array}$$ (5)

where *g*_*Na*_ is the conductance density for Na^+^-ions, *m* and *h* are the gating variables (see Equations 8–10), *E*_*Na*_ is the reversal potential of Na^+^ and *V* is the membrane potential. This fast ion channel is needed to reproduce the fast rising phase of T-cell action potentials. An M-type potassium channel was added to cease spiking during current injection (Benda and Herz, 2003; Meiser et al., 2019). It was formulated as:


$$\begin{array}{c} I_{M}=g_{M}\cdot z^{2}\cdot \left(E_{K}-V\right) \end{array}$$ (6)

where *g*_*m*_ is the maximum conductance of the M-type channel and *z* is the gating variable (see Equations 8–10). The leak current *I*_*L*_ was formulated as follows:


$$\begin{array}{c} I_{L}=g_{L}\cdot E_{L} \end{array}$$ (7)

where *g*_*L*_ is the leak conductance and *E*_*L*_ is the reversal potential of the leak current. Calcium dependent potassium channels are also present in leech T cells but reportedly act on the timescale of minutes rather than seconds (Baylor and Nicholls, 1969). Since our simulations occur over time periods in the range of seconds, we decided not to include these types of ion channels in order to simplify our model. The contribution of voltage-dependent Ca^+^ conductances is negligible (Valkanov and Boev, 1988).

Kinetic equations for the fast and slow potassium channels (see Equations 3, 4) were formulated as:


$$\begin{array}{c} \frac{dx\left(t\right)}{dt}=\frac{x_{\infty }\left(V\right)-x}{\tau_{x}} \end{array}$$ (8)

Where *x* is either *n*_*f*_, *n*_*s*_, *p*, *m*, *h*, or *z*, *x*_∞_ corresponds to the respective steady state value and τ_*x*_ is the respective time constant. The steady-state functions in Equation 8 were defined by:


$$\begin{array}{c} x_{\infty }\left(V\right)=\frac{1}{1+e^{\left(-\frac{V+x_{1}}{x_{2}}\right)}} \end{array}$$ (9)

For the sodium and M-type potassium current (see Equations 5, 6, and8), τ_*x*_ was voltage dependent and was calculated as:


$$\tau_{x}(V)=T_{x}\cdot (\frac{2}{e^{\left(-\frac{V+x_{1}}{x_{2}}\right)}+e^{\left(\frac{V+x_{1}}{x_{2}}\right)}})$$ (10)

The parameters for the kinetic equations are listed in Table 2 and were not varied in the parameter study. Minor changes in ion channel kinetics can greatly impact the simulated response, but a systematic study would exceed the scope of the present paper. Instead, the parameters for the ion channel kinetics of K^+^-channels were approximated from patch clamp experiments as stated above. The kinetics for Na^+^-channels were manually fitted to reproduce the characteristic spike shape of fast T-cell spikes.

**Table 2:** Constant parameter values of ion-channel kinetics.

Current	Unit	Parameter	Value	Corresponding equations
*I*_*K*_*f*__	mV	*n*_*f*_1__; *n*_*f*_2__	1; 16.5	9
*I*_*K*_*f*__	mV	*p*_1_; *p*_2_	15; 8	9
*I*_*K*_*f*__	ms	τ_*n*_*f*__	1	8
*I*_*K*_*f*__	ms	τ_*p*_	39.41	8
*I*_*K*_*s*__	mV	*n*_*s*_1__; *n*_*s*_2__	25; 29.19	9
*I*_*K*_*s*__	ms	τ_*n*_*s*__	67.18	8
*I*_*M*_	mV	*z*_1_; *z*_2_	40.76; 2.62	9, 10
*I*_*M*_	ms	*T*_*z*_	94.03	10
*I*_*Na*_	mV	*m*_1_; *m*_2_	20; 11	9, 10
*I*_*Na*_	mV	*h*_1_; *h*_2_	28; 10.35	9, 10
*I*_*Na*_	ms	*T*_*m*_	0.8	10
*I*_*Na*_	ms	*T*_*h*_	19.25	10

Although many studies have investigated the somatic membrane currents of leech T cells, they have not provided a quantitative analysis of ion channel distributions (Valkanov and Boev, 1988; Stewart et al., 1989; Baylor and Nicholls, 1969; Jansen and Nicholls, 1973). Therefore, we compiled parameter ranges from manually tested parameter sets and ensured that at least one valid response was found for each tested parameter value for our test morphology. The T-cell model published by Meiser et al. (2023) was used as a starting point for manually exploring the parameter space. To simulate a spike initiation zone, we separated sections of the reconstructed morphologies (Figures 1B, F) into two classes: active and passive. We set different parameter ranges for *g*_*Na*_, *g*_*K*_*f*__ and *g*_*M*_, respectively (see Table 1). The active section contained higher parameter values for *g*_*Na*_ to simulate a spike initiation zone (Kole et al., 2008). To ensure that action potentials are generated in the SIZ and not in the passive compartments, we varied the parameter values for *g*_*Na*_ in the passive compartments to a lower range. Simulating T-cell-typical fast repolarization periods required high conductances for *g*_*K*_*f*__ in active and passive sections. Values for *g*_*M*_ were systematically lower in active sections to facilitate spike generation. *g*_*K*_*s*__was introduced with small conductance densities to adjust afterhyperpolarization durations. The conductance density remained the same in the active and passive compartments. *E*_*L*_ and *g*_*L*_ were adjusted to fit subthreshold response features, such as the resting membrane potential and input resistance. The ranges of the tested parameter values were set based on manual testing. We varied the ranges of these parameters within range of values, often used in leech cell models (Cataldo et al., 2005; Lamb and Calabrese, 2013; Kretzberg et al., 2007). To our knowledge, the K^+^ and Na^+^ reversal potentials of leech T cells have not yet been published and were therefore varied in the parameter study.

### 4.4 Spike initiation zone configurations

In the standard configuration, the SIZ was located in the root process of the 1RP morphologies. For the 2RP morphologies, both root processes contained a SIZ with the same conductance density. Hence, the total conductance was higher in the standard configuration of 2RP than of 1RP morphologies. In the “one SIZ distal” configuration in Figure 6, the SIZ in the distal root process had the same conductance density as in the standard configuration, while the membrane properties of the proximal root process were set to the passive parameter settings. In the “two SIZs distal” configuration in Figure 6, the conductance density in the distal root process was increased to match the total conductance of both SIZs in the standard configuration, while the proximal root process again contained passive membrane. For the homogeneous configurations “hom” in Figures 5, 6 we calculated the total conductance of *g*_*K*_*f*__, *g*_*M*_ and *g*_*Na*_ in the standard configuration and distributed it homogeneously over the membrane surface. For the “hom one SIZ” configuration in Figure 6, we used the “one SIZ distal” configuration and distributed its total conductance homogeneously.

### 4.5 Parameter study

As described above for the experimental data, we simulated responses to a shortened stimulation protocol for somatic current injection. To evaluate a response for a given parameter set, we compared each response feature to the corresponding data derived from the experiment. We considered a simulated response “valid” if the following inequation was true for all analyzed response features:


$$\begin{array}{c} min\left(x_{i}\right)<{\tilde{x}}_{i}<max\left(x_{i}\right) \end{array}$$ (11)

where *max*(*x*_*i*_) and *min*(*x*_*i*_) denote the maximum and minimum of experimentally measuered response feature values and ${\tilde{x}}_{i}$ as the corresponding response feature value in the simulated response.

Using the Python package psweep, we created a parameter grid with all possible combinations of the given parameters (Table 1). The sweeps were parallelized on the HPC cluster of the University of Oldenburg, utilizing the Dask Python package (Dask Development Team, 2016). The parameter sweeps were done in two steps: 1: All combinations of the parameters in Table 1 were applied to one T-cell morphology with one branch. The simulated responses were compared to the experimentally observed responses concerning the response features listed above. 2: The parameter combinations that yielded valid responses for the selected example anatomy were tested on the entire population of morphologies. In this parameter search, a parameter set was considered valid if inequation 11 was true for all morphologies. For the examples presented in Section 2.1, we decided to show a morphology with response feature values at the center of the response feature distribution for the entire population. For the example simulations in the “Results” section, we randomly selected three parameter sets that yielded different spike counts, mimicking highly, moderately and less active T cells in our data set (Table 1) and validated that the resulting spike feature values also matched the experimental pairwise joint distributions of all response features (Supplementary Figure 3).

## Data Availability

The datasets presented in this study can be found in online repositories. The names of the repository/repositories and accession number(s) can be found below: https://gin.g-node.org/Strandpalme/Sandbote_et_al_2026.
